# The impact of emotional content on pseudoword recognition

**DOI:** 10.1007/s00426-020-01454-6

**Published:** 2020-12-18

**Authors:** Simone Sulpizio, Eleonora Pennucci, Remo Job

**Affiliations:** 1 Department of Psychology, University of Milano-Bicocca, Piazza dell’Ateneo Nuovo, 1, 20126 Milan, Italy; 2 Department of Psychology and Cognitive Science, University of Trento, Corso Bettini, 84, 38068 Rovereto, Italy

## Abstract

The present study investigates the influence of emotional information on language processing. To this aim, we measured behavioral responses and event-related brain potentials (ERPs) during four Italian lexical decision experiments in which we used emotionally intense and neutral pseudowords—i.e., pseudowords derived from changing one letter in a word (e.g., *cammelto*, derived from *cammello* ‘camel’ vs. *copezzolo*, from *capezzolo* ‘nipple’)—as stimuli. In Experiment 1 and 2, half of the pseudowords were emotionally intense and half were neutral, and were mixed with neutral words. In Experiment 3, the list composition was manipulated, with ¼ of the pseudowords being derived from emotionally intense words and ¾ derived from neutral words. Experiment 4 was identical to Experiment 1, but ERPs were recorded. Emotionally intense pseudowords were categorized more slowly than neutral pseudowords, with the difference emerging both in the mean and at the leading edge of the response times distribution. Moreover, emotionally intense pseudowords elicited smaller N170 and N400 than neutral pseudowords. These results speak in favor of a fast and multi-level infiltration of the emotional information into the linguistic process of word recognition.

## Introduction

All mammals share the ability to recognize and react to emotional content since it is crucial for survival. Empirical research on animals and humans has repeatedly shown that emotionally salient stimuli facilitate visual perception (e.g., Phelps & Carrasco, 2006), drive attention (e.g., Khalid, Horstmann, Ditye, & Ansorge, 2017; Öhman, Flykt, & Esteves, 2001), and affect learning (e.g., Hu et al., 2007; Morris, Öhman, & Dolan, 1998). Differently from all mammals, the human being has the faculty of language, which is massively used for communication: Thanks to language, we can easily share our thought about ourselves and the physical and psychological world.

Despite the language being among our privileged means to communicate emotional content, the research on whether and how emotion modulates language processing is scanty. The interest in the relation between language and emotion has increased recently (e.g., Dhooge & Hartsuiker, 2011; Estes & Adelman, 2008; Grecucci, Sulpizio, Tommasello, Vespignani, & Job, 2019; Kousta, Vinson, & Vigliocco, 2009; Sulpizio, Grecucci, & Job, 2020; Sulpizio, Toti, Del Maschio, Costa, Fedeli, Job, & Abutalebi, 2019). The results of these studies show that emotion words differ from neutral words and that the valence of the emotion words has a role, but the directions of the effects are not consistent. Here, we focus on the issue from a different perspective: We investigate the extent to which the emotional content of words may modulate the early-orthographic and late-semantic stages of stimulus encoding occurring during lexical decision. To this aim, we ran a series of experiments collecting both behavioral and event-related potentials (ERPs) data using pseudowords.

Most of the behavioral and ERP evidence on emotional words refers to the semantic dimension, which is manipulated to investigate the word's ability to trigger emotional reactions. At the behavioral level, in a lexical decision study, Kuperman, Estes, Brysbaert, and Warriner (2014) reported that emotional dimensions—as expressed by continuous measures of both valence and arousal—exerted a monotonic effect on word processing: The greater the negativity and the higher the arousal, the slower were the lexical decision times. Moreover, valence and arousal did not interact with each other. Still, both interacted with word frequency, exerting a larger effect on low- than on high-frequency words: According to the authors, this pattern suggests that the emotional effect arises at, and can thus affect, a late (i.e., lexico-semantic) stage of stimulus encoding. At the ERP level, most of the literature has focused on the modulation of the two components typically associated with emotional processing, i.e., early posterior negativity (EPN) and late positive potential (LPP; for an extensive review, see Citron, 2012), which are informative on the emotional content of the stimulus independently of its modality (i.e., words, pictures, sounds). However, the available literature is silent on whether the emotional content may affect the hardwired linguistic processes: There is little and contrasting evidence on whether emotional content may modulate those components that are a marker of word processing, as the N170 which is a marker of orthographic differentiation (e.g., it differentiates words form nonwords; Carreiras, Armstrong, Perea, & Frost, 2014; Maurer, Rossion, & McCandliss, 2008), and thus indexes an early stage of processing that occurs during the encoding of the printed stimulus—and the N400—which is a marker of lexical access and lexico-semantic processing (Kutas & Federmeier 2011; Lau, Phillips, & Poeppel, 2008), and thus indexes a later stage of processing during stimulus encoding.

In a lexical decision study, Scott, O'Donnel, Leuthold, and Sereno (2009) reported that word frequency and emotionality interacted by modulating the amplitude of N170: For low-frequency words, the N170 was larger for neutral than for positive or negative words; instead, for high-frequency words, the N170 was larger for negative than for positive or neutral words. The findings were interpreted as indicating that emotional content affects the early-orthographic stages of processing. Differently, in a recent study, Grecucci et al. (2019) asked participants to rate visually presented emotional (negative) and neutral words on valence and arousal and failed to report any N170 modulation. These results do not support the hypothesis of an early-orthographic effect of emotional content. Similarly, Kissler et al. (2007) asked participants to read emotional (pleasant, unpleasant) and neutral words. They found that the effect of emotional content—starting at 200 ms and visible on the posterior sites—had an opposite direction than that reported by Scott et al. (2009), with emotional stimuli showing a larger negativity than neutral stimuli. The authors concluded that “the ERP enhancement to emotional stimuli occurs on the level of semantic analysis, not at a prelexical stage” (p. 479, see also Palazova, Mantwill, Sommer & Schact, 2011). This conclusion is also supported by studies reporting that emotional content modulates the N400 (e.g., Kanske & Kotz, 2007; Scott et al., 2009).

While there is a large consensus that the emotional content affects lexico-semantic processing, whether it may also modulate early stages of processing as, for example, orthographic processing, is still an open issue since the available evidence is scanty and inconclusive. To shed light on this issue, we ran a series of lexical decision experiments in which we manipulated the emotional content using pseudowords orthographically similar to real words. Specifically, we constructed pseudowords derived either from neutral or from emotionally intense words by changing only one letter (e.g., *cammelto*, derived from *cammello* ‘camel’ vs. *copezzolo* derived from *capezzolo* ‘nipple’).

The novel and daring move of using pseudowords to investigate the effects of the emotional content on language processing stems from two inter-related considerations. First, pseudowords can activate their base words without participants being readily and/or necessarily aware of such activation (Ferrand & Grainger, 1994). Thus, we may be able to detect the effects of the emotionality of words limiting the effects of “surprise” or defense mechanisms effects we might expect upfront. Second, since the pseudoword (e.g., *copezzolo*) and not the word (i.e., *capezzolo* ‘nipple’) is the to-be-processed target, this would yield a further advantage, by potentially limiting the effect that emotionally intense words may have on subsequent trials since emotionally intense words are never presented throughout our experiments (e.g., Algom, Chajut, & Lev, 2004). Such a research strategy has been used to address semantic processing (e.g., Bentin, McCarthy, & Wood, 1985; Smith & Halgren, 1987; Ziegler, Besson, Jacop, Nazir, & Carr, 1997) and, in the way we use it here, may shed light on how emotional aspects of the word are processed.

On the assumption that a pseudoword activates orthographically similar words, including the corresponding base word, we may expect that activation of the base words may be a function, among other things, of the emotional content of the words. If it is the case that emotional words are activated more rapidly than neutral words (Kousta et al., 2009), information about the emotional content of the words should be available sooner and would be more salient for these words than for neutral words. This, in turn, should feedback to the pseudoword, enhancing its possible status as a word, because of the strong semantic activation: To reject as a candidate word an emotionally intense pseudoword, the system has to recruit more information to contrast strong information both at the orthographic and the semantic level. Cast in terms of models of word recognition, our predictions can be outlined in the following way. In a lexical decision task, the “no” response is given by means of a flexible-deadline criterion that is set up on the activation accumulated at the orthographic stage: A higher activation in early processing (i.e., orthographic lexicon) would increase the deadline, with a consequent increase in response times and (possibly) accuracy (e.g., Coltheart, Davelaar, Jonasson, & Besner, 1977; Coltheart, Rastle, Perry, Langdon, & Ziegler 2001; Grainger & Jacobs, 1996). The deadline account is clearly exemplified by the pseudoword frequency effect—pseudowords derived from a high-frequency word take longer to be categorized as nonwords than pseudowords derived from a low-frequency word (e.g., Perea, Rosa, & Gomez, 2005)—and the pseudoword neighorhood size effect—pseudowords with many similar spelled words are responded more slowly and more accurately than those with few similarly spelled words (e.g. Coltheart et al., 1977; Forster & Shen, 1996). Following this logic, by using neutral and emotionally intense pseudowords we should be able to directly investigate whether the emotional content may affect the early-orthographic stage of stimulus encoding (for semantic effects on early orthographic processing in word recognition, see, e.g., Chen, Davis, Pulvermuller, & Hauk, 2015; Wang, Deng, & Booth, 2019). By not being blurred by the influence of emotional words, pseudowords may provide unique information on the early processes of lexical decision, during which lexical processes are used to generate signals to perform the word/pseudoword discrimination (Yap, Sibley, Balota, Ratcliff, & Rueckl, 2015). In this perspective, pseudowords may help to investigate the impact of emotional content on early and late processes occurring during the encoding of a printed stimulus. Thus, pseudowords allow us to better distinguish between early orthographic processing—in which information about letters and other orthographic features (e.g., letter combinations) is used to compute the orthographic word identity—and late lexico-semantic processing—in which the available information is used to access the word meaning. The use of pseudowords may also be of particular importance in our ERP experiment to increase the chance to detect modulations of language-sensitive components (i.e., N170, N400) without the risk of overlooking them with word-specific, emotion-related components (i.e., EPN, LPP).

The present study comprises four lexical decision experiments. In all experiments, filler neutral words were presented together with emotionally intense (e.g., *copezzolo*) and neutral pseudowords (e.g., *cammelto*). In Experiment 1 and 2, the ratio of emotionally intense and neutral pseudowords was 1:1. If emotional content impacts on lexico-semantic processing and percolates into orthographic processing, we expect response times to emotional pseudowords being longer than those to neutral pseudowords. To collect evidence on the task processing stage at which the emotional effects arise in the decisional process—which we hypothesize to be during the stimulus encoding, not the following decisional stage—, we inspected the means of the RT distributions but also their leading edge (0.1 quantile), which is related to the rate with which information is accumulated and to the quality of information derived from the stimulus (e.g., Ratcliff, Gómez, & McKoon, 2004). Since fast responses may be affected mostly by early activation processes (this is the case, e.g., of the deadline mechanism on the basis of early lexical activation), the analysis of the leading edge may be informative about the stage at which a variable may affect the processing (for the same logic and analysis, see, e.g., Perea et al., 2005). In particular, if the activation of emotional content may impact on the early stages of stimulus encoding, with the difference between emotional and neutral pseudowords arising already during early lexical processing, then such difference should be visible also in the edge of the distribution. This prediction follows from the logic that the deadline mechanism for the “no” response in the lexical decision depends on the activation at the early stages of processing. If the difference between emotionally intense and neutral pseudowords emerges later on in the task processing, for example, during the decisional stage (e.g., Perea et al., 2005), no effect in the leading edge of the distribution should be detected. In Experiment 3, we manipulated the list context so that the ratio of emotional intense and neutral pseudowords was 1:3. This contextual variation allowed us to test the robustness of the effect, and, more important, to what extent it is susceptible to strategic behaviors (e.g., Forster & Sheen, 1996; Monsell, Patterson, Graham, Hughes, & Milroy, 1992). Finally, in Experiment 4, we replicated the first experiment by recording ERPs to unveil the exact temporal dynamic of the effects of the emotional content on the word recognition process: If emotional content impacts on lexico-semantic processing and percolates into the orthographic processing, then modulation of the N170 can be expected, with smaller N170 for emotionally intense than neutral pseudowords: A similar result would suggest that emotion does interfere with the recognition system early on. However, if emotions play a role only late during stimulus encoding, i.e., when semantic analysis occurs, ERP effects are expected only from the N400 onward, with smaller N400 for emotionally intense than neutral pseudowords.

## Experiment 1

### Method

#### Participants

Twenty-nine students (24 females, mean age: 20.82, sd: 1.64) from the University of Trento took part in the experiment as volunteers. They were all Italian native speakers with normal or corrected-to-normal vision. The study—as well as all the following ones—was approved by the ethical committee of the University of Trento. The datasets generated and/or analyzed during the current and the following experiments are not publicly available because we did not obtain consent for publication from the participants. Data are available from the corresponding author on reasonable request.

#### Materials

Two sets of 80 pseudowords each were created by changing one letter from existing words—a vowel was always replaced with another vowel, and a consonant with another consonant. Replacements were always phonotactically legal and were analogous, both in terms of position and type (vowel/consonant), in the two sets. One set was derived from emotionally intense words belonging to the categories of sexuality and illness, which are among the categories eliciting the strongest emotional reactions (e.g., Bradley & Lang, 2007). The other set was derived from neutral words belonging to the categories of objects and animals. For pseudowords creation, the first and the last letter of the word were never changed. To be sure that these pseudowords could activate the base word from which they were derived from, a pre-test was run: Twenty-one university students (13 female, mean age: 21.3 sd: 2.61) were involved in a paper-and-pencil task in which they were presented with the full list of pseudowords and were asked to write the word from which each pseudoword was derived. For each pseudoword, we then calculated the percentage of recognition (i.e., the number of correct identifications divided by the maximum number of possible recognitions) and excluded those that were recognized less than 50% of times. After this selection we had two sets of 70 pseudowords each that were balanced on: percentage of recognition, frequency of the base word, letter and syllable length, orthographic neighbourhood size, orthographic neighbours’ summed frequency, Orthographic Levenshtein Distance (OLD), and bigram frequency (all ps > 0.08). The two sets, however, differed for valence and arousal, with emotionally intense pseudowords being derived from more negative and arousing words (henceforth, emotionally intense pseudowords) than pseudowords derived from neutral words (henceforth, neutral pseudowords) (both ps < 0.001; Table 1). One-hundred and forty words were selected as fillers and were balanced with pseudoword on: letter and syllable length, orthographic neighbourhood size, orthographic neighbours’ summed frequency (all *p *> 0.1). No emotionally intense word was included among the fillers. All stimuli are listed in the Appendix.

**Table 1:** Summary statistics: means (and standard deviations) for the stimuli used in Experiment 1

Item variables	Pseudoword type			
	Experiment 1, 2 and 4		Experiment 3	
	Emotionally intense	Neutral	Emotionally intense	Neutral
Percentage of recognition of the base word	86.32 (12.31)	89.31 (10.70)	86.54 (13.50)	90.00 (9.38)
Frequency of the base word	2.33 (0.54)	2.46 (0.70)	2.45 (0.60)	2.31 (0.65)
Valence of the base word	3.67 (0.77)	5.65 (1.44)	3.53 (1.51)	5.77 (0.60)
Arousal of the base word	5.51 (0.62)	2.89 (0.75)	5.66 (0.58)	2.86 (0.80)
Letter length	7.51 (1.39)	7.42 (1.44)	7.37 (1.23)	7.50 (1.56)
Syllable length	3.21 (0.67)	3.05 (0.58)	3.12 (0.64)	3.12 (0.60)
N of orthographic neighbors	1.24 (0.69)	1.35 (0.68)	1.36 (0.76)	1.35 (0.66)
Neighbors’ frequency	6.03 (12.30)	6.40 (12.73)	7.39 (15.42)	4.47 (5.06)
OLD	2.22 (0.52)	2.07 (0.44)	2.15 (0.49	2.16 (0.50)
Bigram frequency	11.49 (0.45)	11.39 (0.40)	11.49 (0.36)	11.35 (0.41)

Logarithmic frequency of the base word is extracted from SUBTLEX.IT (freely available at http://crr.ugent.be/subtlex-it/; Crepaldi, Keuleers, Mandera, & Brysbaert, 2013). Valence and arousal have been collected in a rating including all the stimuli used in the present study; each stimulus was judged by 15 participants for each dimension

#### Procedure

Participants sat at a distance of about 50 cm from the screen. Each trial started with a fixation cross, presented for 300 ms in the center of the screen and was followed by a blank for 200 ms. Then, a stimulus appeared in the same position and was presented until the participant’s response or for a maximum of 2000 ms. The interstimulus interval was 1000 ms. Participants were tested individually. They were asked to indicate, as quickly and accurately as possible, whether each letter string was a real word or not. Responses were given by pressing either X or M on the keyboard. The response button was counterbalanced across participants. Stimuli were presented in 2 blocks. The order of stimuli was randomised within blocks and block order was counterbalanced between participants. A set of 8 practice trials preceded the experiment. The experiment was run using the E-Prime Software (Psychology Software Tools, Pittsburgh, PA, USA; www.pstnet.com). After the end of the experiment, participants were given a sheet containing all the pseudowords used in the experiment and were asked to write next to each pseudoword the base word from which it was derived. The aim of this manipulation check was to ensure that, when looking at pseudowords, all participants tended to activate the base words.

## Results and discussion

In this and the following experiments, the main analysis focused on pseudowords, which were the target of the study. Before analysing them, however, an ancillary analysis was run on all stimuli to verify the presence of the lexicality effect that would guarantee a different processing of words and pseudowords. Data were analyzed by means of mixed-effects models. Analyses were performed using the library lmerTest (Kuznetsova, Brockhoff, & Christensen, 2017) in the software R (R Core Team, 2016). Following Bates, Kliegl, Vasishth, & Baayen’s (2015) suggestion against a maximal random-effects approach, in this and the following experiments, for the random effects part of the models we considered the by-participants and by-items random intercepts. In this and the following experiments the number of observations per cell is enough to ensure a properly powered repeated-measure experiment (see Brysbaert & Stevens, 2018).

One participant was excluded from all analyses because of a low level of accuracy (below 2.5 standard deviations from the overall participants’ mean).

### Ancillary analysis—all stimuli

A linear model was run with RTs as dependent variable and Stimulus lexicality (word vs. pseudoword) as a predictor. The model showed that words were recognized faster than pseudowords (745 ms vs. 882 ms, *β* = − 133.40, st. err. = 11.27, *df* = 271.72, *t* = − 11.84, *p* < 0.001). The logistic model on response accuracy as dependent variable showed no effect (93.85% and 93.59% for words and pseudowords, respectively, *β* = 0.13, st. err. = 0.19, *z* < 1, *p* > 0.4).

### Main analysis—Pseudowords only

For the manipulation check, for each item we calculated the percentage of post-experiment recognition (number of correct identifications divided by the maximum number of possible recognitions). Overall, there was a very high percentage of recognition (88%), which indicated that, when dealing with pseudowords, participants were likely to activate also their corresponding base words.

Results from the lexical decision task are reported in Table 2. To examine RT distributions, we used participants correct RTs to estimate five quantile RTs: the 0.1, 0.3, 0.5, 0.7, and 0.9 quantiles (see, e.g., Perea, Rosa, & Gomez, 2005; Ratcliff et al., 2004; for a similar procedure). Correct RTs, 0.1 quantile, and accuracy were analyzed by means of mixed-effects models. The Type of pseudoword (emotionally intense vs. neutral) was entered as a predictor. The correctness of base word recognition was entered as nuisance covariate (it was entered as a binary measure (1 = correct, 0 = incorrect) at the trial level—this allowed us to account for the fact that a given item did or did not activate the corresponding base word in a given participant).^1^

**Table 2:** RTs for correct responses and percentage of accuracy by condition (with standard deviations), in Experiment 1

	Pseudoword	
	Emotionally intense	Neutral
Mean RTs	904 (166)	859 (149)
0.1 Quantile	662 (113)	641 (111)
Accuracy	94.7 (2.4)	92.5 (6.3)

The linear model on RTs as dependent variable showed that neutral pseudowords were recognized faster than emotionally intense pseudowords (*β* = − 47.48, st. err. = 13.68, *df* = 136.00, *t* = − 3.47, *p* < 0.001). The same pattern also emerged when only data in the 0.1 quantile were analyzed (*β* = − 19.26, st. err. = 4.04, *df* = 53.60, *t* = − 4.76, *p* < 0.001).

The logistic model on response accuracy as dependent variable showed no effect (*z* = − 1.05, *p* > 0.2).

The present results showed slower responses to emotionally intense than neutral pseudowords, and this difference emerged both in the mean and in the leading edge of the RT distribution. These findings would suggest that during word recognition, (intense) emotional content is quickly available within the system and may affect the early stages of stimulus encoding, possibly by increasing the activation accumulated within the orthographic lexicon, and thus the timing to release a “no” response. Before further interpreting our results, more data are desirable to test the replicability of the effect and to better characterize its nature. We, therefore, ran two further experiments, with the aim to (a) establish the stability of the pattern, by running an exact replication of Experiment 1 with a new sample of participants (Experiment 2); (b) investigate the robustness of the effect, by running an experiment in which the list composition is manipulated to contrast for allegedly strategic behaviors (Experiment 3).

## Experiment 2

### Method

#### Participants

Thirty-four students (13 females, mean age: 22.32, sd: 2.67) from the University of Trento took part in the experiment as volunteers. They were all Italian native speakers with normal or corrected-to-normal vision. None participated in the previous experiment.

#### Materials and procedure

Same as in Experiment 1.

## Results and discussion

No participant was excluded from analysis because of low accuracy.

### Ancillary analysis—all stimuli

The linear model on RTs as dependent variable and Stimulus lexicality (word vs. pseudoword) as predictor showed a large lexicality effect, with words being recognized faster than pseudowords (788 ms vs. 927 ms, *β* = − 147.76, st. err. = 11.37, *df* = 272.15, *t* = − 12.99, *p* < 0.001). In addition, the logistic model on response accuracy as dependent variable showed an effect of lexicality, with higher accuracy for words than pseudowords (94.39% and 91.34% for words and pseudowords, respectively, *β* = 0.70, st. err. = 0.16, *z* = 4.27, *p* < 0.001).

### Main analysis—Pseudowords only

As in the previous experiment, for each participant we calculated the percentage of recognition of the original words from the pseudowords. Overall, recognition was very high (96.82%), which indicated that, when processing the pseudowords, the likelihood that participants activated also their corresponding base words was high.

Results from the lexical decision task are reported in Table 3. Correct RTs, 0.1 quantile, and accuracy were analyzed by means of mixed-effects models.

**Table 3:** Mean RTs for correct responses and percentage of accuracy by condition (with standard deviations), in Experiment 2

	Pseudoword	
	Emotionally intense	Neutral
RTs	948 (199)	907 (194)
0.1 Quantile	676 (124)	667 (123)
Accuracy	91.30 (9.76)	91.38 (10.96)

The linear mixed-effects model on RTs as dependent variable included the Type of pseudoword (emotionally intense vs. neutral) as fixed factor and by-participants and by-items random intercepts. The correctness of the base word recognition was included as nuisance fixed factor. The effect of Type of pseudoword was significant (*β* = − 39.52, st. err. = 15.08, *df* = 135.00, *t* = − 2.62, *p* = 0.009), with shorter RTs for neutral than for emotionally intense pseudowords. The same pattern also approached significance in the analysis of the 0.1 quantile (*β* = − 9.72, st. err. = 5.23, *df* = 95.30, *t* = − 1.86, *p* = 0.06).

The logistic mixed-effects model on response accuracy as dependent variable (including the same fixed and random structure of the model on RTs) showed no significant effect (*β* = 0.01, st. err. = 20, *z* < 1, *p* > 0.9).

The findings of Experiment 2 replicate those of the previous experiment, by convincingly showing slower responses to emotionally intense than to neutral pseudowords, both in the mean and in the leading edge of the RTs distribution.

Both Experiment 1 and 2 show the same pattern of longer RTs to pseudowords derived from emotionally intense words than to pseudowords derived from neutral words. As it is well known that participants may strategically control the way they perform the task and that different effects may be modulated by the nature of the list context (e.g., Forster & Sheen, 1996; Grainger, Spinelli & Ferrand, 2000; Logan & Zbrodoff, 1979; Monsell et al., 1992; Perea & Rosa, 2002), we should rule out that the difference between the two types of pseudowords might have been magnified by the list composition as the use of an equal proportion of emotionally intense and neutral pseudowords might have enhanced participants’ attention to emotional stimuli and thus encouraged them to strategically capitalize on this information to accomplish the task’s goal. To rule out this possibility and to test the robustness and generalizability of the effect we reported in the first two experiments, we ran a further experiment in which we diluted the overall strength of the emotional list by increasing the number of neutral pseudowords (and filler words). In the new list, the pseudowords were for ¼ derived from emotional intense words and for ¾ derived from neutral words. If the difference between emotionally intense and neutral pseudowords was mainly due to a strategic optimization of the decision process, Experiment 3 should show no difference between the two conditions. Otherwise, the pattern of Experiments 1 and 2 should be replicated.

## Experiment 3

### Method

#### Participants

Twenty-seven students (24 females, mean age: 21.44, sd: 2.11) from the University of Trento took part in the experiment as volunteers. They were all Italian native speakers with normal or corrected-to-normal vision. No one had participated in the previous experiments.

#### Materials

Eighty pseudowords were selected from Experiment 1 (40 emotionally intense and 40 neutral pseudowords, equally divided between the two conceptual categories for each class of stimuli; stimuli are listed in Appendix). The two sets were balanced on: percentage of recognition; frequency of the base word, letter and syllable length, orthographic neighbourhood size, orthographic neighbours’ summed frequency, OLD, and bigram frequency (ps > 0.1; Table 1); the two sets differed for valence and arousal, with emotionally intense pseudowords being more negative and arousing than neutral pseudowords (both ps < 0.001; Table 1). Moreover, to create the list manipulation, 80 additional neutral pseudowords and 20 words (for a total of 160 pseudowords and 160 words) were added as fillers. The additional pseudowords were derived from words belonging to the two categories used in Experiment 1 (i.e., objects and animals) by replacing one letter (firs and last letter were never changed). None of the new filler word was emotionally salient.

#### Procedure

The same as in Experiment 1 and 2.

## Results and discussion

No participant was excluded from analysis because of low accuracy.

### Ancillary analysis—All stimuli

The linear model on RTs as dependent variable and Stimulus lexicality (word vs. pseudoword) as predictor showed a large lexicality effect, with words being recognized faster than pseudowords (748 ms vs. 880 ms, *β* =  −  129.03, st. err. = 11.66, *t* = − 11.06, *p* < 0.001). The logistic model on response accuracy as the dependent variable showed no effect of lexicality (92.60% and 93.01% for words and pseudowords, respectively, *z* < 1, *p* > 0.7).

### Main analysis—Pseudowords only

For the manipulation check, there was a very high percentage of recognition of the original words the pseudowords were derived from (93%), which indicated that when processing pseudowords it was highly likely that participants activated also their corresponding base words.

Results from the lexical decision task are reported in Table 4. Correct RTs, 0.1 quantile RTs, and accuracy were analyzed by means of mixed-effects models with participants and items as random intercepts and Type of pseudoword (emotionally intense vs. neutral) as a fixed factor. The correctness of base word recognition was entered as a nuisance covariate.

**Table 4:** Mean RTs for correct responses and percentage of accuracy by condition (with standard deviations), in Experiment 3

	Pseudoword	
	Emotionally intense	Neutral
RTs	898 (156)	855 (133)
0.1 Quantile	638 (100)	617 (86)
Accuracy	94.25 (5.31)	91.66 (6.27)

The linear mixed-effects model on RTs as dependent variable showed that neutral pseudowords tended to be responded faster than emotionally intense pseudowords (*β* = − 38.27, st. err. = 21.96, *df *= 76.80, *t* = − 1.74, *p* = 0.08). The effect was fully significant in the analysis of the 0.1 quantile (*β* = − 22.36, st. err. = 6.10, *df* = 179.66, *t* = − 3.66, *p* < 0.001).

The logistic model on response accuracy as dependent variable showed no significant effect (*β* = 0.31, st. err. = 0.34, *z* < 1, *p* > 0.3).

### Between experiments analysis

To test whether the list context manipulation modulated the effect we reported, a further linear mixed-effects model was run with RTs as dependent variable and Type of pseudowords (emotionally intense vs. neutral) and Experiment (1 plus 2 vs. 3) as fixed factors. The correctness of base word recognition was entered as a nuisance variable. The analysis showed a main effect of Type of pseudowords (*β* = − 41.21, st. err. = 14.00, def = 144.00, *t* = − 2.94, *p* = 0.003), with neutral pseudowords being faster than emotionally intense pseudowords. The main effect of Experiment approached significance (*β* = − 15.95, st. err. = 8.67, def = 10,027.00, *t* = − 1.83, *p* = 0.06): participants tended to be faster in Experiment 3 than in the previous experiments. The interaction was not significant (*β* = − 14.38, st. err. = 12.23, def = 10,019.00, *t* = − 1.17, *p* > 0.2). To quantify the amount of evidence for the null interaction, Bayes Factor was calculated using the BayesFactor package (version 0.9-12-2, Morey & Rouder, 2015) with the model including the Type of pseudowords × Experiment as the denominator and the model without the interaction as the numerator. The Bayes factor was 31.75, indicating very strong evidence for the null interaction.

The same pattern also emerged when data of the 0.1 quantile were analysed: The main effect of Type of pseudowords was significant (*β* = − 18.95, st. err. = 4.24, *t* = − 4.46, *p* < 0.001); no further effect reached significance (both ts < 1, ps > 0.3). The Bayes Factor calculated with the model including the interaction as the denominator and the model without the interaction as the numerator was 7.63, indicating moderate evidence for the null interaction.

The results clearly indicate that the list context manipulation did not affect the processing of emotionally intense pseudowords.

Overall, the results of Experiment 3 nicely replicate those of the first two experiments, by showing slower responses—both in the mean and at the leading edge of RTs distribution—to emotionally intense than neutral pseudowords. Moreover, and more importantly, the between-experiments analysis showed that the manipulation of list composition did not modulate the effect, providing evidence for its generalizability and suggesting that the activation of the base word and its emotional content is not dampened by strategies use.

The present findings offer strong evidence that the activation of intense emotional content may affect stimulus processing early on during word recognition. The emergence of the effect in the 0.1 quantile of RTs distribution is in accordance with a view of lexico-semantic information affecting early (possibly orthographic) stages of stimulus encoding. To collect more cogent evidence in favor of such interpretation we ran Experiment 4, in which we replicated Experiment 1 with the addition of the ERP recording. In this way, we were able to track the time dynamic of the behavioral effect and unveil the stage(s) of processing at which emotionally intense content exerts its effect. If it impacts on early orthographic processing, we expect to see a modulation of N170. If instead, only lexico-semantic analysis is sensitive to the emotional information, our manipulation should modulate the N400 component, but no effect on the earlier ERP components should be visible.

## Experiment 4

### Method

#### Participants

Twenty-six students (16 females, mean age: 22, sd: 2.22) from the University of Trento took part in the experiment as volunteers. All participants were right-handed, Italian native speakers; they had normal or corrected-to-normal vision and reported to be neurologically healthy. Participants gave written informed consent to their participation after they were completely informed about the nature of the study. No one had participated in one of the previous experiments.

#### Materials

Same as Experiment 1.

#### Procedure

Each trial started with a fixation cross, presented for 300 ms in the center of the screen; the fixation was followed by a short blank for 200 ms; then a stimulus appeared in the same position and was presented until participant’s response or for a maximum of 2000 ms. Finally, a blink cue (–|–) was presented for 2500 ms and was followed by a short inter-stimulus interval for 300 ms; participants were asked to blink only when such blink cue was presented. A brief practice (8 stimuli) preceded the experiment. All the other details were identical to those of Experiment 1. The experiment was run using E-Prime software (version 2.0, Psychology Software Tools, Pittsburgh, PA, USA; www.pstnet.com).

#### EEG recording and analyses

EEG was recorded using a BrainAmp DC amplifier (Brain Products GmBH) from 25 scalp electrodes (Fp1, Fp2, Fz, F3, F4, F7, F8, F9, F10, Cz, FC5, FC6, C3, C4, T7, T8, Pz, CP5, CP6, P3, P4, P7, P8, O1, O2) mounted on an elastic cap (BrainCap by Brain Products GmBH), positioned according to the international standard position (10–20 system). Additional external electrodes were placed on mastoids (A1, A2) and below (Ve1, Ve2) the eyes. All sites were referenced to the left mastoid (A1) and the ground was placed in the Afz site. Impedance was kept below 10 kΩ. Data were acquired at the sampling rate of 250 Hz with a low-pass filter with 100 Hz cutoff frequency and 10 s time constant.

To better detect blinks and ocular movements, two virtual EOG channels were off-line computed as the difference between the average of Fp1 and Fp2 and the average of Ve1 and Ve2 (VEOG), and as the difference between F9 and F10 (HEOG). The continuous signal was corrected for eye blinks and ocular movements by using Independent Component Analysis (ICA algorithm: Infomax) and re-referenced to the average mastoids activity. Data were low-pass filtered (20 Hz cutoff, 12 dB/oct) and a high pass filtered (0.05 Hz cutoff 12 dB/oct). Channels Ve1, Ve2, A1, A2, VEOG, and HEOG were excluded from statistical analyses.

EEG was segmented up to 800 ms after target onset. Artifact rejection was performed by means of an automatic threshold rejection algorithm: Epochs at which the voltage exceeded [− 100 μV, 100 μV] for any site were not included in the average. Because of the large number of epochs contaminated by artifacts, two participants remained with few epochs (2.5 SD below the mean) and were thus discarded. In addition, trials where participants gave a wrong or no response were rejected from the dataset before averaging. Overall, 21.8% of the trials were excluded before ERP average (9.2% because of wrong or no response and 12.6% because of artifacts, a number in line with that reported in the lexical decision literature (e.g., Carreiras et al., 2007; Scott et al., 2009); number of excluded trials for type of stimuli: Emotional intense pseudowords: *M* = 21.06%, SD = 4.92%, MIN = 12.5%, MAX = 32.5%; neutral pseudowords: *M* = 22.58%, SD = 4.61%, MIN = 13.8%, MAX = 32%). Single-subject waveforms for each condition were averaged in reference to the 100 ms pre-target baseline.

## Results

Due to the low accuracy (below 2.5 SD from the overall mean), one participant was discarded from all analyses.

### Behavioral results.

#### Ancillary analysis—all stimuli

The linear model on RTs as dependent variable and Stimulus lexicality (word vs. pseudoword) as predictor showed a large lexicality effect, with words being recognized faster than pseudowords (747 ms vs. 874 ms, *β* = − 126.56, st. err. = 9.97, *df* = 270.94, *t* = − 12.69, *p* < 0.001). Also the logistic model on response accuracy as dependent variable showed an effect of lexicality (92.80% and 90.80% for words and pseudowords, respectively, *β* = 0.32, st. err. = 0.16, *z* = 1.97 1, *p* = 0.04).

#### Main analysis—Pseudowords only

Data are reported in Table 5. Correct RTs, 0.1 quantile RTs, and response accuracy were analyzed by means of mixed-effects models with participants and items as random factors, and Type of pseudoword (emotionally intense vs. neutral) as factor. The correctness of the base word recognition was entered as nuisance covariate.

**Table 5:** Mean RTs for correct responses and percentage of errors by condition (with standard deviations), in Experiment 4

	Pseudoword	
	Emotionally intense	Neutral
RTs	881 (119)	869 (114)
0.1 Quantile	660 (79)	650 (80)
Accuracy	91.6 (6.05)	90 (5.54)

When looking at the mean of the distribution, the linear mixed-effects model on RTs failed to show a significant effect of Type of pseudoword (*β* = − 12.20, st. err. = 11.59, *t* = − 1.05, *p* > 0.2). However, the pattern of RTs mirrored that obtained in all previous experiments—i.e., faster RTs for neutral than emotionally intense pseudowords. It is possible that the ERP procedure—in which participants were instructed to avoid any unnecessary movement—might have contributed to the thinning of the behavioral effect. To further investigate the absence of a significant effect at the presence of a numerical difference, and to quantify the amount of evidence for the null effect, we calculated the Bayes Factor with the model including the effect of Type of pseudoword and the model without it as the numerator; the Bayes Factor was 1.09, indicating no evidence for any of the sides. Because of the inconclusiveness of this result, we calculated the confidence intervals (CI) for the model’s parameter estimated for the Type of pseudoword in Experiment 1 and 2 (which were identical to the present experiment in both stimuli and list context) and compared these CI with the parameter estimated for the same predictor in the present analysis. The results are the following: Experiment 1 CI [− 18.63, −76.11], Experiment 2 CI [− 9.86, − 69.16]. These values show a large variability, with the lower boundary that can assume relatively small values, which include (in Experiment 2) or are close to (in Experiment 1) the parameter estimated in the present experiment (i.e., − 12.20). This suggests that, although small, the ~ 12 ms difference reported in Experiment 4 is a plausible size for the effect of Type of pseudoword.

The effect of Type of pseudoword was significant in the analysis of 0.1 quantile, with neutral pseudowords being faster than emotionally intense pseudowords (*β* = − 10.31, st. err. = 4.75, *df* = 101.800, *t* = − 2.17, *p* = 0.03).

The logistic model on response accuracy did not show any significant effect (*β* = − 0.08, st. err. = 0.23, *z* < 1, *p* > 0.7).

### ERPs results

#### N170

Three pairs of electrodes (P3/4, P7/8, and O1/2) from the left and right posterior areas of the scalp were selected since these regions are those with maximal N170 effects (e.g., Maurer, Brandeis, & McCandliss, 2005). The mean amplitude in the 150-200 ms time window was calculated for the above sites (e.g., Bentin, Mouchetant-Rostaing, Giard, Echallier, & Pernier, 1999; Dien, 2009; Maurer et al., 2005; Yum, Holcomb, & Grainger, 2011; Zhang, Su, Chen, Ng, Wang, & Yang, 2020), and submitted to a 2 (Type of pseudoword: emotionally intense vs. neutral) × 2 (hemisphere: right vs. left) ANOVA with all factors as within-participants.

**Fig. 1:**
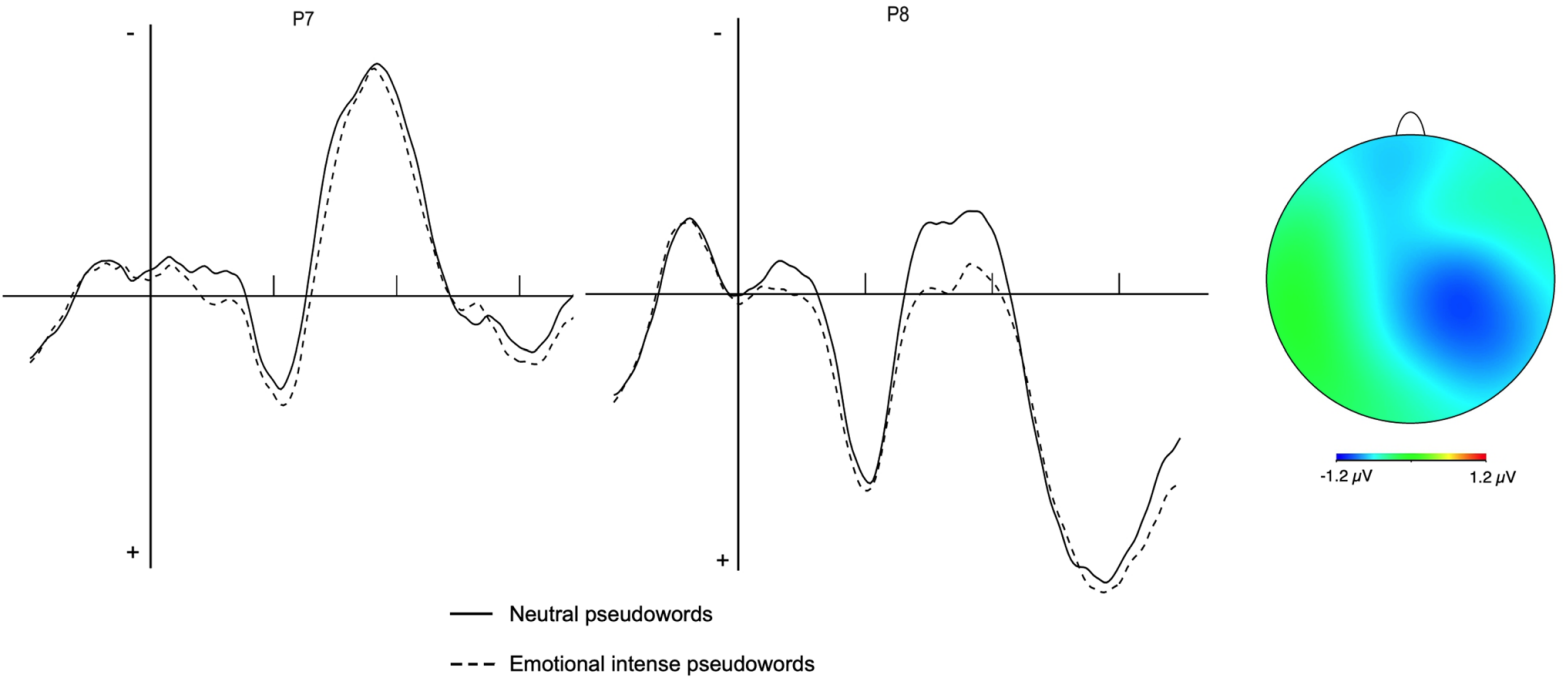
ERP waveforms (**a**) for emotional and neutral pseudowords at P7 and P8 and voltage maps (**b**) centered on the measurement epochs for the N170; the map was calculated by subtracting the neutral from the emotional intense condition. In subplot **a**, ERPs are time-locked to the target onset; the three short ticks indicate 100 ms intervals; negative voltages are plotted up

The main effect of Type of pseudoword was not significant (*F* (1, 23) = 2.76, *p* > 0.1, η^2^_G_ = 0.007). However, the two-way interaction was significant (*F* (1, 23) = 7.86, *p* = 0.01, η^2^_G_ = 0.001). The inspection of the interaction, by splitting the data for the factor Hemisphere, showed that the effect of Type of pseudoword was significant for the left (*F* (1, 23) = 5.36, *p* = 0.02, η^2^_G_ = 0.01), but not for the right hemisphere (*F* (1, 23) = 0.87, *p* > 0.4, η^2^_G_ = 0.002). The main effect of Hemisphere was significant (*F* (1, 23) = 7.10, *p* = 0.01, η^2^_G_ = 0.03), with a larger negativity on the left than on the right hemisphere (Fig. 1).

#### N400

**Fig. 2:**
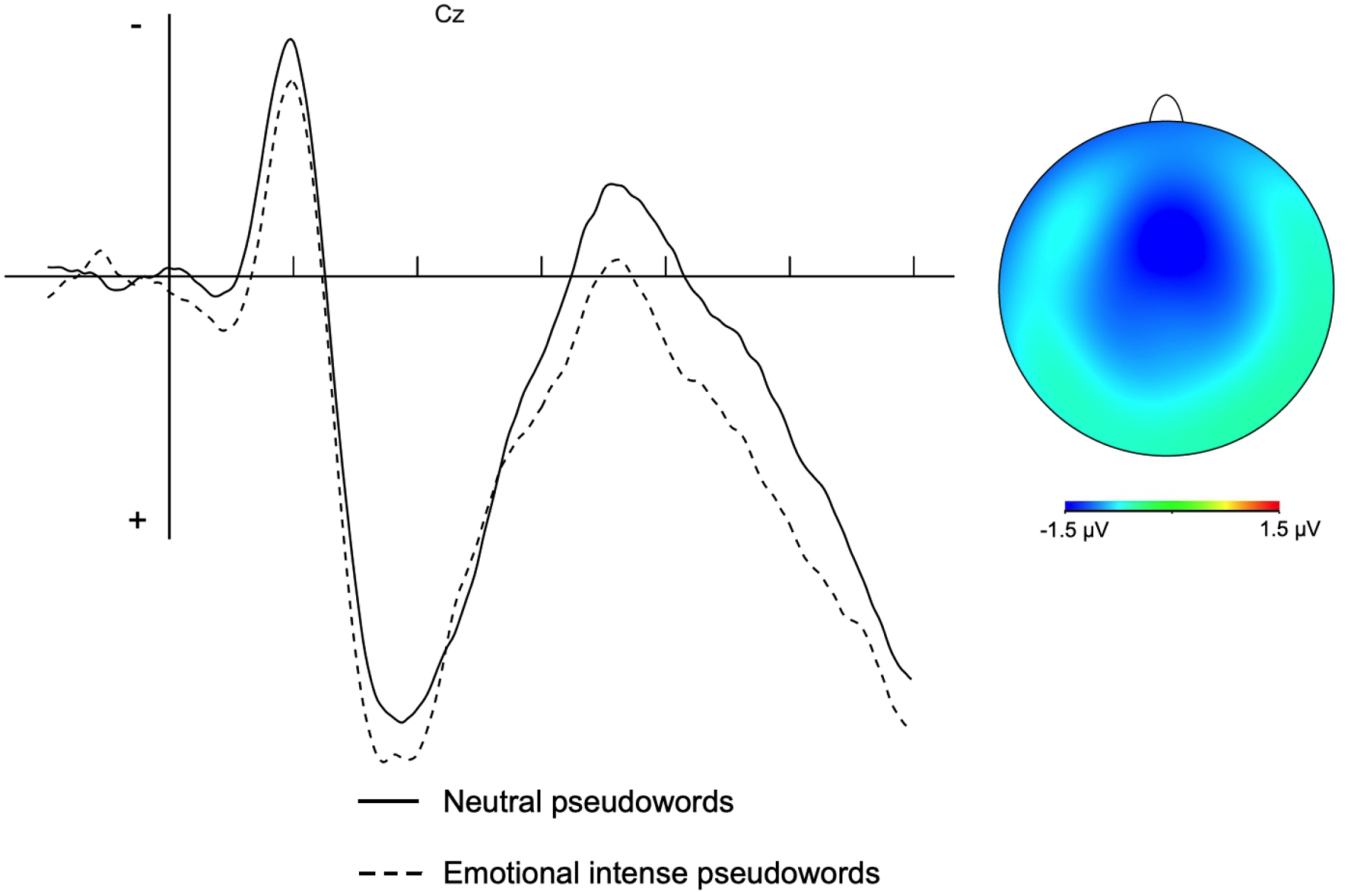
ERP waveforms (**a**) for emotional and neutral pseudowords at a representative electrode (Cz) and voltage maps (**b**) centered on the measurement epochs for the N400; the map was calculated by subtracting the neutral from the emotional intense condition. In subplot **a**, ERPs are time-locked to the target onset; the short ticks indicate 100 ms intervals; negative voltages are plotted up

The N400 was analyzed both at lateral and midline sites, since this component was broadly distributed on the entire scalp. To this aim, nine electrodes (F3, Fz, F4, C3, Cz, C4, P3, Pz, P4) were defined as the Electrode factor; the mean amplitude in the 300–500 ms time window was calculated for the above sites (e.g., Carreiras, Vergara, & Perera, 2007; Holcomb, 1993; Lau et al., 2008; Sulpizio & Job, 2018), and submitted to a 2 (Type of pseudoword: emotionally intense vs. neutral) × 3 (Hemisphere: Right vs. Central vs. Left) × 3 (Longitude: Frontal vs. Central vs. Posterior) ANOVA with all factors as within-participants. The Geisser and Greenhouse (1959) correction was applied if needed (only corrected p values are reported). The analysis showed a main effect of Type of pseudoword (*F* (1, 23) = 12.95, *p* = 0.001, η^2^_G_ = 0.01), with emotionally intense pseudowords being less negative than neutral pseudowords (see Fig. 2). Also topographic factors were significant (Longitude: *F* (2, 46) = 30.31, *p* < 0.001, η^2^_G_ = 0.1; Hemisphere: *F* (2, 46) = 6.24, *p* = 0.004, η^2^_G_ = 0.01; Longitude × Hemisphere: *F* (4, 92) = 9.23, *p* < 0.001, η^2^_G_ = 0.007, with positivity increasing toward the posterior sites and especially for the central line). No further effect reached significance (Type of pseudoword × Longitude: *F* (2, 46) = 1.86, *p* > 0.1, η^2^_G_ = 0.0001; Type of pseudoword × Hemishpere: *F* (2, 46) = 1.67, *p* > 0.1, η^2^_G_ = 0.0001; Type of pseudoword × Longitude × Hemisphere: *F* (4, 92) = 1.12, p > 0.3, η^2^_G_ = 0.00005).

The data clearly show a two-wave pattern, with an N170 and an N400, indicating early and late effects of emotional content on word recognition.

## General discussion

The present study aimed to investigate whether and to what extent emotional content may affect different stages of linguistic processing, in particular early orthographic and late lexico-semantic processing stages. The issue was tested in four lexical decision experiments in Italian, in which our core manipulation involved pseudowords that were derived from emotionally intense (e.g., *copezzolo,* base word ‘nipple’) and neutral words (e.g., *cammelto*, base word ‘camel’). Across experiments, behavioral results showed that participants were slower in responding to emotional than neutral pseudowords. At the electrophysiological level, the two types of pseudoword diverged at both early-orthographic and late-semantic stage of processing, as reflected by the modulation of N170 and N400, respectively. Taken together, our results invite two main conclusions: (a) during word recognition, emotional content affects several loci of the process; (b) the effects of the emotional content triggered by the pseudowords are generalizable and arise early during processing. In what follows, we will discuss the empirical evidence for our proposal. Moreover, the similarity between the effects elicited here by emotional pseudowords with those typically elicited by real emotional words suggests that the processing of these pseudowords relies on the activation of their base word. Before starting the discussion, however, it is important to acknowledge some limitations concerning the effect we reported. Our emotionally intense pseudowords were derived from two categories—i.e., illness and sexuality. Although there are no a priori reasons to assume critical differences among different stimulus categories, the generalizability of our findings to all emotional words is an empirical question for future research.

The finding that emotionally intense and neutral pseudowords differed from one another both in the mean and at the leading edge of the RT distribution represents a first hint on the timing of the effect. Since fast responses are mostly affected by early task processes related to the stage of stimulus encoding (e.g., Perea et al., 2005), the shift in the leading edge we reported would suggest that the manipulation of the pseudowords' emotional content affects this early stage of word recognition.

A further and more direct evidence that the emotional content modulates the orthographic processing characterizing the early encoding of the stimulus comes from our first ERP effect that occurred between 150 and 200 ms after target presentation, with neutral pseudowords showing a larger negativity than emotionally intense ones. For its timing and topography—i.e., posterior sites—the effect can be interpreted as an N170 modulation, which is an early marker of visual recognition. Interestingly, while linguistic stimuli usually elicit a left-lateralized N170 modulation (e.g., Hauk, Patterson, Woollams, Watling, Pulvermuller, & Rogers, 2006; Sereno, Reyner, & Posner, 1998), the difference we found was evident in the right hemisphere. A tentative explanation may be sketched by considering that while for emotional content a right prevalence has been reported in the literature (e.g., Ladavas, Nicoletti, Umiltà, & Rizzolatti, 1984; LeDoux, 1995), this advantage may be visible also in studies investigating language processing. For example, Scott et al. (2009) used a lexical decision task with emotional words and reported a larger bilateral N170 for neutral than for (positive and negative) emotional low-frequency words. Fruholtz et al. (2011) evaluated the effect of the emotional content of words using an implicit task in which participants were presented with colored emotional and neutral words and faces, and were asked to name the color. In their analysis of the right N170 for words, the authors found that it was affected by the emotional content of the stimuli, possibly influencing processing in the extrastriate regions. A right hemispheric advantage for emotional words has also been reported by Ortigue et al. (2004): These authors used emotional and neutral words in a lexical decision task with ERP recording while presenting pairs of stimuli bilaterally, one to the right and one to the left visual field. By analyzing scalp topographies and their brain sources, Ortigue and colleagues found that responses to the right visual field were better characterized by a scalp topography yielding bilateral occipital sources with current density maximum at the right hemisphere. Since all other conditions were mainly associated with a similar left-hemisphere source, the authors concluded that the words emotional content activates a network in the right hemisphere. This network would be active since the early stages of visual processing, and it would trace the emotional value of the stimulus. Note that, although visuo-orthographic processing occurs mainly in the left hemisphere, recent neuroimaging evidence suggests a bilateral visuo-orthographic processing that proceeds in parallel up to the left and right visual word-form area, which would show a symmetrical interhemispheric connectivity among them (Chu & Meltzer, 2019; Rauscheker, Bowen, Parvizi, & Wandell, 2012; for a right-hemisphere orthographic processing, see also the literature on patients with deep dyslexia or global alexia, e.g., Bonandrini et al., 2020; Coltheart, 2000; Larsen, Baynes, & Swick, 2004; Weekes, Coltheart, & Gordon, 1997). Therefore, in our experiment, emotional information (which is preferentially processed in the right hemisphere) might have initially interacted with the right hemisphere visuo-orthographic processing.

As for the generalizability of the process, we point out that the same effect occurred across different experiments, with different lists of pseudowords and different ratios between neutral and emotional pseudowords (i.e., the same effect occurred independently of the proportion of emotionally intense pseudowords included in the experiment), speaking in favor of an effect that is driven by the stimulus processing itself more than on the strategies use encouraged by the list context. This pattern suggests that the activation of the emotional content may impact on the recognition process even when not directly available to the beholder's eyes: It is only by means of the early and fast activation of the base word that the semantic content may interfere with the pseudoword processing. This is in line with the view that the recognition of an orthographic string, independently of its lexical status, is mediated by both the lexical and the sublexical procedure (e.g., Coltheart et al., 2001; Job, Peressotti, & Cusinato, 1998).

If at least part of the effect(s) of emotionally intense pseudowords arises during -orthographic processing, what are the mechanisms responsible for the effect? We propose that emotional content exerts its effect within the orthographic lexicon by modulating its activation level. Since neutral and emotionally intense pseudowords were equated for orthographic similarity, the different activation level for the two types of pseudowords must be due to the speed with which the corresponding base words are activated and their meaning accessed. The account we put forward is that the emotional content enhances activation of the base word through the joint effects of orthographic and semantic processing in an interactive activation fashion. The joint action of rapidly activated emotional and orthographic information renders the emotionally intense pseudowords more word-like than neutral pseudowords, rendering the recognition process more difficult.

This account can be framed in the “deadline criterion” framework. According to this proposal, a “no” response in a lexical decision task is given by means of a deadline criterion that considers the activation accumulated within the orthographic lexicon (e.g., Coltheart et al., 2001; Grainger & Jacobs, 1996; Lupker, Brown, & Colombo, 1997). The response deadline is flexible and a higher activation in the orthographic lexicon would increase the deadline, i.e., the participant needs more time to say “no” to a pseudoword that triggers stronger orthographic processing. This is what happens, for example, in experiments involving high- and low-frequency pseudowords, in which pseudowords derived from high-frequency words take longer to be categorized as pseudowords than pseudowords derived from low-frequency words (e.g., Marcolini, Burani, & Colombo, 2009; Perea et al., 2005). In a similar vein, in our study emotionally intense pseudowords elicited higher orthographic activation, which in turn made the “no” decision harder.

In order for our account to hold, it is crucial that the emotional content has an early impact on word recognition. Our results show a clear effect of the emotional content on the initial stages of pseudoword processing. The comparative result for the mean and leading hedge, the null effect of list composition, and the modulation of the N170 suggest that once available, emotional information—an inherent semantic feature of the stimulus—percolates into the early stages of word recognition and affects orthographic processing. Converging evidence for this interpretation comes from a recent fMRI study by Sulpizio et al. (2019), who ran a lexical decision experiment with taboo and neutral words. The authors found that, compared to neutral words, taboo words were associated to a weaker involvement of the left and right fusiform gyrus, a structure specialized in the extraction and storing of abstract patterns from visual(-orthographic) information. The result suggests that semantic information, via top-down processing, may affect visual-orthographic processing, with taboo words being more word-like—and thus requiring less visual-orthographic processing—than neutral words. A similar conclusion is in line with the proposal by Windmann et al. (2002), who argued that, when a stimulus is visually presented, emotional significance may be evaluated even before the word is fully recognized.

Our results showed a second ERP effect that, for its timing and topography, we interpreted as an N400 modulation. The N400 component is considered an index of the process of lexical access and lexico-semantic integration (e.g., Lau et al., 2008). A modulation of N400 in response to emotional stimuli has been reported by Kanske and Kotz (2007). In their study, the authors tested the effects of word concreteness (abstract vs. concrete) and emotionality (positive vs. negative vs. neutral valence) in a lexical decision study. Their results showed smaller N400 for negative than neutral words, a pattern indicating facilitated processing for emotional with respect to neutral stimuli. This interpretation may also hold for the pattern we obtained, with the neutral pseudowords triggering a more demanding semantic processing than emotionally intense pseudowords. As an index of lexico-semantic processing, however, the N400 is also sensitive to the lexical status of the stimulus and has been reported to be larger for pseudowords than words (e.g., Bentin, 1987; Kissler & Herbert, 2013). It may be argued that, as a peculiar semantic dimension, emotionality may increase the lexical status of emotional pseudowords, rendering them more word-like, which would thus elicit a smaller N400 for these stimuli than for the neutral ones. In other words, being associated with an emotional content increases, ceteris paribus, the probability of a written stimulus to be a word. Therefore, the difference in the N400 amplitude between emotional intense and neutral pseudowords might have a double origin, i.e., the easiness of processing emotional content and/or the lexical status of the stimuli.

Although we argue that emotional content affects pseudoword recognition through base word activation, an alternative interpretation might be suggested. Recently, using distributional semantics, Hendrix and Sun (2020) showed that it is possible to compute pseudoword meaning on the basis of letter n-grams (i.e., letter substrings) without passing through lexical information. The authors also investigated the effects of semantic measures associated with pseudowords on lexical decision and found that pseudowords that are semantically more similar to real words are rejected more slowly than those that are more dissimilar. These findings would suggest that pseudowords are not semantically empty and that their visual presentation drives activation in the semantic system similar to that driven by real words (for a similar approach and conclusions, see Cassani et al., 2020; Chuang et al., 2020). Interestingly, the authors claim that “the more similar the activation patterns in the semantic system for a nonword are to the activation patterns in the semantic system for real words, the more word-like a nonword is” (Hendrix & Sun, 2020, p. 23). In such a perspective, it may be tempting to interpret our effects as a consequence of the direct activation in the semantic system elicited by the pseudowords—which would then affect the processing of orthographic information too—without assuming any base word mediation. However, the design of our experiment was not aimed at testing such hypothesis and thus prevents us from drawing any conclusion about it.

To conclude, our results show behavioral—which are visible both in the mean and at the leading edge of the RT distribution—and ERP evidence that emotional content may quickly intrude on the processing of word recognition since its early stages, even when indirectly evoked by means of pseudowords. This pattern indicates that emotional information may exert a direct influence at multiple stages on language processing, percolating into both the visuo-orthographic and lexico-semantic processing.

## Stimuli used in Exp. 1, 2 and 4

*Emotional intense pseudowords (and base words).* Aloressia (anoressia, *anorexia*), ampresso (amplesso, *intercourse*), asna (asma, *asthma*), astrite (artrite, *arthritis*), azeurisma (aneurisma, *aneurysm*), bronchete (bronchite, *bronchitis*), caglione (coglione, *idiot*), cappelta (cappella, *cock-up*), carcimoma (carcinoma, *carcinoma*), castrapo (castrato, *castrated*), cerrosi (cirrosi, *cirrhosis*), clitonide (clitoride, *clitoris*), coico (coito, *coitus*), colela (colera, *cholera*), copezzolo (capezzolo, *nipple*), corite (colite, *colitis*), culastone (culattone, *fag*), debenza (demenza, *dementia*), diabede (diabete, *diabetes*), diorrea (diarrea, *diarrhea*), distrufia (distrofia, *dystrophy*), ebbolia (embolia, *embolism*), eccifato (eccitato, *arroused*), emorroivi (emorroidi, *hemorroids*), epatife (epatite, *hepatitis*), epolessia (epilessia, *epilepsy*), ereziome (erezione, *erection*), ermia (ernia, *slipped disc*), eropismo (erotismo, *eroticism*), fraltura (frattura, *fracture*), fricio (frocio, *faggot*), genitani (genitali, *genital*), infarzo (infarto, *heart attack*), ingulata (inculata, *con*), irtus (ictus, *ictus*), isterea (isteria, *hysteria*), libibo (libido, *libido*), liucemia (leucemia, *leukemia*), maniapo (maniaco, *maniac*), mataria (malaria, *malaria*), megnotta (mignotta, *whore*), meningete (meningite, *meningitis*), minghia (minchia, *dick*), molessia (molestia, *teasing*), ninfobane (ninfomane, *nymphomaniac*), orcasmo (orgasmo, *orgasm*), polmocite (polmonite, *pneumonia*), pornocrafia (pornografia, *porhography*), prepufio (prepuzio, *foreskin*), psigosi (psicosi, *psychosis*), pumpino (pompino, *blowjob*), purtaniere (puttaniere, *someone who goes whoring*), puttona (puttana, *bitch*), sadomavo (sadomaso, *sadomasochist*), salsonella (salmonella, *salmonella*), sclerusi (sclerosi, *scleroris*), scopaza (scopata, *fuck*), sfarlattina (scarlattina, *scarlatina*), sgroto (scroto, *scrotum*), sifilite (sifilide, *syphilis*), sperna (sperma, *sperm*), stubro (stupro, *rape*), testigolo (testicolo, *testicle*), teteno (tetano, *tetanus*), tumole (tumore, *tumor*), ulcema (ulcera, *ulcer*), uteno (utero, *uterus*), vagena (vagina, *vagina*), vaiogo (vaiolo, *smallpox*), vebratore (vibratore, *dildo*).

*Neutral pseudowords.* Ariede (ariete, *ram*), azino (asino, *donkey*), baleba (balena, *whale*), bostiglia (bottiglia, *bottle*), cammelto (cammello, *camel*), candena (candela, *candel*), caprialo (capriolo, *roe deer*), cavarletta (cavalletta, *grasshoper*), chidarra (chitarra, *guitar*), coccoddillo (coccodrillo, *crocodile*), colnice (cornice, *frame*), conillio (coniglio, *rubbit*), coscino (cuscino, *pillow*), cricedo (criceto, *hamster*), cufria (cuffia, *bonnet*), delsino (delfino, *dolphin*), diorio (diario, *diary*), diveno (divano, *sofa*), drocedario (dromedario, *dromedary*), etefante (elefante, *elephant*), faciolo (fagiolo, *bean*), fiascola (fiaccola, *torch*), fizzoletto (fazzoletto, *handkerchief*), forgica (formica, *ant*), gabbiado (gabbiano, *seagull*), ganzella (gazzella, *gazelle*), gazedo (gazebo, *gazebo*), ghepando (ghepardo, *cheetah*), giriffa (giraffa, *giraffe*), gorilca (gorilla, *gorilla*), guvo (gufo, *owl*), ilbuto (imbuto, *funnel*), istrige (istrice, *porcupie*), labirunto (labirinto, *labirinth*), lavanna (lavagna, *blackboard*), lucertula (lucertola, *lizard*), modile (mobile, *forniture*), mormotta (marmotta, *marmot*), nuvota (nuvola, *cloud*), ombrollo (ombrello, *umbrella*), oschio (occhio, *eye*), pacora (pecora, *sheep*), pappapallo (pappagallo, *parrot*), pavote (pavone, *peacock*), pilcino (pulcino, *chick*), piramite (piramide, *pyramide*), piscima (piscina, *swimmingpool*), piumeno (piumino, *quilted jacket*), pneusatico (pneumatico, *tire*), poccione (piccione, *pidgeon*), prisna (prisma, *prism*), righelco (righello, *ruler*), rubbica (rubrica, *telephone book*), salnone (salmone, *salmon*), scirmia (scimmia, *monkey*), sciurpa (sciarpa, *scarf*), scoialtolo (scoiattolo, *squirrel*), scorfione (scorpione, *scorpion*), scualo (squalo, *shark*), tampuro (tamburo, *drum*), tappavella (tapparella, *roller shutter*), tapreto (tappeto, *carpet*), telefoco (telefono, *telephone*), uzignolo (usignolo, *nithingale*), volanze (volante, *flying*), volvola (valvola, *valve*), zalzara (zanzara, *mosquito*), zaspa (zappa, *hoe*), zedra (zebra, *zebra*), zettera (zattera, *raft*).

## Stimuli used in Exp. 3

*Emotional intense pseudowords.* Aloressia (anoressia, *anorexia*), ampresso (amplesso, *intercourse*), caglione (coglione, *idiot*), cappelta (cappella, *cock-up*), carcimoma (carcinoma, *carcinoma*), cerrosi (cirrosi, *cirrhosis*), colela (colera, *cholera*), corite (colite, *colitis*), debenza (demenza, *dementia*), diabede (diabete, *diabetes*), epatife (epatite, *hepatitis*), epolessia (epilessia, *epilepsy*), eropismo (erotismo, *eroticism*), fricio (frocio, *faggot*), infarzo (infarto, *heart attack*), irtus (ictus, *ictus*), isterea (isteria, *hysteria*), liucemia (leucemia, *leukemia*), maniapo (maniaco, *maniac*), mataria (malaria, *malaria*), megnotta (mignotta, *whore*), minghia (minchia, *dick*), ninfobane (ninfomane, *nymphomaniac*), orcasmo (orgasmo, *orgasm*), pornocrafia (pornografia, *pornography*), psigosi (psicosi, *psychosis*), pumpino (pompino, *blowjob*), puttona (puttana, *bitch*), sclerusi (sclerosi, *sclerosis*), scopaza (scopata, *fuck*), sfarlattina (scarlattina, *scarlatina*), sgroto (scroto, *scrotum*), sifilite (sifilide, *syphilis*), sperna (sperma, *sperm*), stubro (stupro, *rape*), testigolo (testicolo, *testicle*), tumole (tumore, *tumor*), vagena (vagina, *vagina*), vaiogo (vaiolo, *smallpox*), vebratore (vibratore, *dildo*).

*Neutral pseudowords.* Ariede (ariete, ram), baleba (balena, whale), caprialo (capriolo. roe deer), chidarra (chitarra, guitar), coccoddillo (coccodrillo, crocodile), coscino (cuscino, pillow), cufria (cuffia, bonnet), diveno (divano, sofa), drocedario (dromedario, dromedary), faciolo (fagiolo, bean), fizzoletto (fazzoletto, handkerchief), forgica (formica, ant), gabbiado (gabbiano, seagull), gazedo (gazebo, gazebo), giriffa (giraffa, giraffe), guvo (gufo, owl), ilbuto (imbuto, fannel), istrige (istrice, porcupie), labirunto (labirinto, labirinth), lavanna (lavagna, blackboard), lucertula (lucertola, lizard), mormotta (marmotta, marmot), nuvota (nuvola, cloud), ombrollo (ombrello, umbrella), pacora (pecora, sheep), pappapallo (pappagallo, parrot), pavote (pavone, peacock), piramite (piramide, pyramide), piscima (piscina, swimmingpool), piumeno (piumino, quilted jacket), pneusatico (pneumatico, tire), righelco (righello, ruler), rubbica (rubrica, telephone book), sciurpa (sciarpa, scarf), scoialtolo (scoiattolo, squirrel), scorfione (scorpione, scorpion), scualo (squalo, shark), tapreto (tappeto, carpet), uzignolo (usignolo, nithingale), zaspa (zappa, hoe).

## Footnotes

1 In this and the following experiments, the analyses were also ran adding the participants' gender as covariate. These analyses showed the same results as those reported.
